# Impact of *in vitro* gastrointestinal digestion on peptide profile and bioactivity of cooked and non-cooked oat protein concentrates

**DOI:** 10.1016/j.crfs.2021.02.003

**Published:** 2021-02-12

**Authors:** Oscar Abel Sánchez-Velázquez, Edith Oliva Cuevas-Rodríguez, Martin Mondor, Sabine Ribéreau, Yves Arcand, Alan Mackie, Alan Javier Hernández-Álvarez

**Affiliations:** a Programa Regional de Posgrado en Biotecnología, Facultad de Ciencias Químico-Biológicas, Universidad Autónoma de Sinaloa, Av. Universitarios s/n, Ciudad Universitaria, Col. Universitarios, P. C. 80030, Culiacán Rosales, Sinaloa, Mexico; b Posgrado en Ciencia y Tecnología de Alimentos, Facultad de Ciencias Químico-Biológicas, Universidad Autónoma de Sinaloa. Av. Universitarios s/n, Ciudad Universitaria, Col. Universitarios, P. C. 80030, Culiacán Rosales, Sinaloa, Mexico; c St-Hyacinthe Research and Development Centre, Agriculture and Agri-Food Canada, 3600, Casavant West Boulevard, Saint-Hyacinthe, Quebec, Canada, J2S 8E3; d Institute of Nutrition and Functional Foods (INAF), Université Laval, Quebec City, Quebec, G1V 0A6, Canada; e School of Food Science & Nutrition, University of Leeds, LS2 9JT, Leeds, United Kingdom

**Keywords:** Oat bioactive peptides, Aqueous-alkaline extraction, *In vitro* gastrointestinal digestion, Amino acids, Antioxidant activity

## Abstract

Oat (*Avena sativa*) is one of the most cultivated and consumed cereals worldwide. Recognized among cereals for its high protein content (12%–24%), it makes it an excellent source of bioactive peptides, which could be modified during processes such as heating and gastrointestinal digestion (GID). This work aims to evaluate the impact of heat treatment on the proteolysis of oat proteins and on the evolution of antioxidant peptide released during *in vitro* static GID, in terms of comparative analysis between cooked oat protein concentrate (COPC) and non-heated oat protein concentrate (OPC) samples. The protein extraction method and cooking procedure used showed no detrimental effects on protein quality. After GID, the proportion of free amino acids/dipeptides (<0.2 ​kDa) reached >40% for both samples (OPC and COPC), thus producing peptides with low molecular weight and enhanced bioactivity. Furthermore, during GID, the amino acid profile showed an increase in essential, positively-charged, hydrophobic and aromatic amino acids. At the end of GID, the reducing power of OPC and COPC increased >0.3 and 8-fold, respectively, in comparison to the non-digested samples; while ABTS^•+^ and DPPH^•^ showed a >20-fold increase. Fe^2+^ chelating capacity of OPC and COPC was enhanced >4 times; similarly, Cu^2+^ chelation showed a >19-fold enhancement for OPC and >10 for COPC. β-carotene bleaching activity was improved 0.8 times in OPC and >9 times in COPC; the oxygen radical antioxidant capacity assay increased 2 times in OPC and >4.7 times in COPC, respectively. This study suggests that OPC after cooking and GID positively influenced the nutritional and bioactive properties of oat peptides. Thus, COPC could be used as a functional food ingredient with health-promoting effects, as hydrothermal treatment is frequently used for this type of cereals.

## Highlights

• A-APE followed by IEP is an ideal alternative for oat protein isolation/concentration.
• Protein quality (IVPD, PDCAAS, AAS, EAAI, BV and PERs) of COPC showed no negative effects after hydrothermal processing.
• OPC and COPC were digested using the INFOGEST protocol.
• Antioxidant and metal chelating properties of COPC peptides is improved with GID.
• OPC and COPC are potential ingredients for the development of functional and/or nutraceutical foods.

## Graphical abstract

**Figure:**
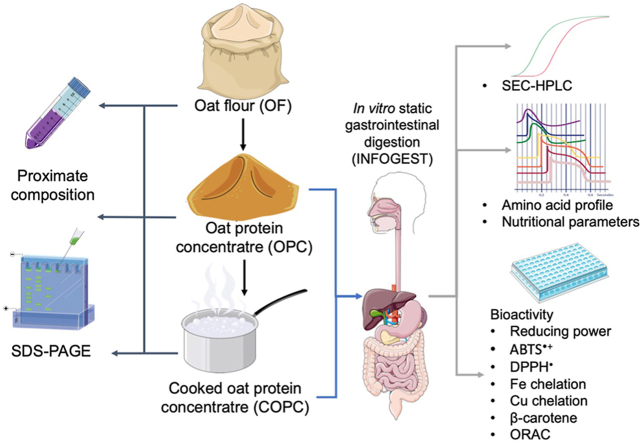

## 1 Introduction

In recent years, the interest in consuming healthy foods has risen. Current's food market demands high-protein or protein enriched ingredients, and this trend has been increasing due to sustainability concerns related to animal proteins production. Thus, causing an increased demand for emerging foods that, in addition to their nutritional value, also exert preventive effects against various health conditions (Boukid et al., 2018). In this way, plant sources, have taken special interest among the population, especially oat; in Canada alone, its consumption has increased from 0.2 to 0.4 million tons in the last three years (USDA-FAS, 2020). Oat (*Avena sativa* L.) is the fifth most-cultivated cereal in the world (Guerrieri, 2018; FAO, 2019), and it is well-known as a source rich in bioactive compounds, including β-glucan, arabinoxylan, oligosaccharides, polyphenols, avenanthramides, indole-gramine alkaloids, flavonolignans, triterpenoid, saponins, sterols and tocols (Cui and Liu, 2014).

In addition to these bioactive compounds, the oat protein content has been reported at between 12.4% and 24.5%, composed mainly of globulins (12%–80%), glutelins (23%–54%), albumins (10%–20%), and avenins (12%–14%). Additionally, oats are considered one of the least allergenic sources of protein (Lásztity, 1996; Klose, 2012; Mäkinen et al., 2017; Kosová et al., 2020). Oat peptides are valuable ingredients with industrial interest for novel food products (Mäkinen et al., 2017; Brückner-Gühmann et al., 2019).

Producing protein concentrates/isolates from oat grain requires methods such as alkaline extraction, isoelectric precipitation, enzymatic hydrolysis (carbohydrases/proteases) and ultrafiltration; thus, in this way starch, fiber and some other non-desirable compounds can be partially and/or completely removed (Mäkinen et al., 2017; Esfandi et al., 2019). Thermal processes, such as extrusion, baking and cooking, are usually employed before the consumption of oat products and thus directly influence the physicochemical, sensory, nutritional and bioactivity of bioactive compounds, especially peptides (Chronakis et al., 2004; Saldanha do Carmo et al., 2019). When heated, oat proteins undergo conformational changes, because the physical forces that favor unfolding increase above those that favor folding. In this way, non-polar amino acids (typically located in the hydrophobic interior of polypeptides) may be exposed to the aqueous phase (Chronakis et al., 2004). Heat treatments may also modify the soluble protein content, monomer-aggregate ratio and relative amino acid distribution of some oat proteins by unfolding, chemical modification and/or reduction of amino acids associated to prolamins and albumins, but also, heating has a selective impact on the different oat protein fractions, for example, increasing hydrophobic amino acid residues of globulins that influence negatively the protein solubility (Guerrieri, 2018; Mäkinen et al., 2017; Gorinstein et al., 1996; Zhao et al., 2004; Runyon et al., 2015). Despite the possible protein denaturation, the high thermal stability of oat proteins suggests that functionality can be retained in heat processed oat products (Gorinstein et al., 1996; Runyon et al., 2015; R Harwalkar and Ma, 1987).

Studies using *in vitro* digestion of oat proteins to mimic gastrointestinal digestion (GID) are reported in the literature, but the heterogeneity of protocols developed has produced discrepancies or conflicting results between research groups on the bioavailability, bioaccessibility, and bioactivity of released peptides (Villemejane et al., 2016; AlHasawi et al., 2018; Feng et al., 2019; Zhong et al., 2019). The multidisciplinary project INFOGEST published a harmonized protocol for a static *in vitro* GID (Minekus et al., 2014; Brodkorb et al., 2019), the results of which have been very similar to those observed *in vivo* for some protein-rich sources under bioethical principles (Tagliazucchi et al., 2016; Hiolle et al., 2020; Sousa et al., 2020). This model has been used to study oat-material macro-compounds, e.g. β-glucan, fiber or lipids (Mackie et al., 2016; Colantuono et al., 2017; Grundy et al., 2017; Korompokis et al., 2018; Rieder et al., 2019), minerals (de Souza et al., 2019), contaminants (Assução et al., 2016) and phytochemicals, such as β-carotene (Zhong et al., 2019), but there is limited research about oat peptides using this model. Therefore, the aim of this work is to evaluate the impact of the heat treatment on the proteolysis of oat proteins and on the evolution of the profile of antioxidant active peptides generated at different stages of static GID under these conditions, in terms of comparative analysis between heated and non-heated oat protein concentrate (OPC) samples.

## 2 Materials and methods

### 2.1 Biological material and reagents

Oat (*Avena sativa* L. var. Navarro) seeds provide by the Modern Research and Development Centre (AAFC) were grown in Brandon, Manitoba, Canada, in 2016 and stored at −20°C. The reagents 2,2′-azino-bis(3-etilbenzotiazoline-6-sulfonicylbenzothiazoline-6-sulphonic acid) (ABTS, A9941), di(phenyl)-(2,4,6-trinitrophenyl)iminoazanio (DPPH, D9132), β-carotene (C9750), 2,2′-azobis(2-amidinopropane), dihydrocloride (AAPH, 440914), potassium sulphate (K_2_S_2_O_8_, 216224), Trolox (238813), Tween® 20 (P7950), linolenic acid (L2376), potassium hexacyanoferrate (III) (K_3_Fe(CN)_6_, P8131), hexahydrated iron(III) chloride (FeCl_3_, 236489), SDS (151-21-3, BioShop®, Canada Inc., Burlington, ON, Canada), β-mercaptoethanol (M3148), calcium chloride (CaCl_2_, 449709), sodium bicarbonate (NaHCO_3_, S6297), D-tryptophan (T-9753), fluorescein (F46960) and protein/peptide standards mix composed of cytochrome c (12 ​kDa, C2436), aprotinin (6.5 ​kDa, A1250000), vitamin B12 (1.3 ​kDa, V2876) and L-carnosine dipeptide (0.2 ​kDa, C9625) were used for size-exclusion chromatography (H2016); enzymes α-amylase from porcine pancreas (EC 3.2.1.1, A3176), pepsin from porcine gastric mucosa (EC 3.24.3.1, P7012), trypsin from porcine pancreas (EC 3.4.21.4, T0303), α-chymotrypsin from bovine pancreas (EC 3.4.21.1, C4129), lipase from porcine pancreas Type II (EC 3.1.1.3, L3126) and bile salt (B8631) were purchased from Sigma-Aldrich (St. Louis, MO, USA), unless otherwise indicated. Laemmli buffer (161–0747), Tris-Tricine (161–0744) and Coomassie Brilliant Blue R-250 (161–0436) solution were supplied by Bio-Rad (BioRad Laboratories, Inc., Mississauga, ON, Canada).

### 2.2 Oat protein concentrate production and cooking process

Oat protein concentrate was produced according to Wu et al. (1977), with some modifications. Raw oat flour (OF) was defatted twice by extraction with hexane (1:5, w/v) for 24 ​h ​at 4°C and then dried under a hood overnight. Defatted OF was mixed with NaOH 0.02 ​N (1:10, w/v) and stirred using a StedFast™ Stirrer (SL 600, Fisher Scientific, Pittsburgh, PA, USA) for 30 ​min ​at pH 9.1 to remove bran and other impurities. The slurry was centrifuged at 9000 ​*g* for 30 ​min ​at room temperature using an Avanti® J-26XPI Centrifuge (Beckman Coulter, Indianapolis, IN, USA). The resulting pellet was treated as per the conditions above, twice. The three recovered supernatants were placed together, and proteins were precipitated at pH 4.0 for 30 ​min and centrifuged as mentioned previously. Supernatant was discarded, and the pellet was washed with distilled water (pH 4.0) and centrifuged using the same conditions (twice). Washed protein precipitate was resuspended in water (1:5, w/v), adjusted to pH 7.0 and freeze-dried. Cooked oat protein concentrate (COPC) was produced as follows: the freeze-dried OPC (1:10, w/v) was dispersed in Millipore water under agitation for 1 ​h ​at 20 ​°C, boiled in a water bath at 100 ​°C for 10 ​min, stored overnight in a freezer at −80 ​°C, freeze-dried in a VirTis model 50- SRC-5 freeze-dryer (VirTis Co., Inc., Gardiner, NY, USA), and then ground using a domestic coffee grinder (model BA-800, Hudson's Bay Co., Toronto, ON, Canada) (Yeoh et al., 2011).

### 2.3 Proximate composition

Proximate nutritional compositions of OF, OPC and COPC were determined using official methods (AACC, 1999). Crude protein was determined by Kjeldahl method with a nitrogen conversion factor of 5.83 (AACC 46-30.01); Fat content was determined using a SER 148 Solvent Extractor (Velp Scientica srl, Milan, Italy) equipped with six Soxhlet posts (AACC 30-25.01). Moisture was also determined by drying the samples overnight at 100 ​°C in a Fisher Isotemp Vacuum Oven (Fisher Scientific Co., Montreal, QC, Canada) (AACC 44-01.01). Ash content was determined by incinerating 1.0 ​g of samples in a muffle oven at 550 ​°C for 12 ​h (AACC 08-16.01). Lastly, total carbohydrate content was calculated by difference. All determinations were done in triplicate, and average values were calculated.

### 2.4 Electrophoresis of proteins (SDS-PAGE)

Electrophoresis of proteins (SDS-PAGE) was performed according to Laemmli et al. (Laemmli, 1970) using a Mini-Protean 3 Gel Electrophoresis Unit (Bio-Rad) and Criterion TGX™ Precast Any kD gel (5671124 Bio-Rad Laboratories, Inc., CA, USA). Samples (OF, OPC and COPC) were dissolved in Laemmli buffer (0.1 ​M Tris-Tricine, pH 6.8, 2% SDS, 5% β-mercaptoethanol and 0.025% bromophenol blue), boiled for 5 ​min, loaded onto the gel (10 ​μL, 20 ​μg protein/well) and run at 150 ​kV. Gel was stained using 0.125% Coomassie Brilliant Blue R-250 in 7% acetic acid and 40% MeOH (v/v) solution and stained in 7% acetic acid and 30% EtOH (v/v) solution. As a molecular marker, Precision Plus Protein™ standard (10–250 ​kDa, Bio-Rad Laboratories Inc., CA, USA) was used.

### 2.5 *In vitro* gastrointestinal digestion

OPC and COPC were digested in three stages (oral, gastric and intestinal phases) according to the harmonized INFOGEST 2.0 protocol of static *in vitro* GID conditions, including enzymes, CaCl_2_ and the simulated oral (SOF), gastric (SGF) and intestinal (SIF) fluids described by Brodkorb et al. (2019), with slight modifications. Briefly, freeze-dried samples were stirred in water (1:2, w/v) and an aliquot of 5 ​mL was taken as a non-digested (ND) aliquot. Food-mix was dissolved in SOF (1:1, v/v) at pH 7.0 to add 75 U of α-amylase/mL and stirred at 37 ​°C for 2 ​min. Then, 5 ​mL was taken as an oral-phase aliquot. To simulate the gastric phase (GP), oral-phase bolus was mixed with SGF (1:1, v/v) at 37 ​°C and adjusted to pH 3 before adding 2000 U of porcine pepsin/mL. Gastric phase (GP) aliquots were taken each 30 ​min for 2 ​h. Afterwards, gastric digested food was dissolved in SIF (1:1, v/v), adjusted pH to 7.0, trypsin (100 U/mL), α-chymotrypsin (25 U/mL), α-amylase (200 U/mg of carbohydrates in sample), lipase (2000 U/mg of fat in sample) and bile salt (10 ​mM) were added to the intestinal phase (IP). Intestinal phase aliquots were taken every 30 ​min for 2 ​h. All aliquots were adjusted to pH 7.0 and heated at 75–80 ​°C for 15 ​min in order to inactivate enzymes.

### 2.6 Size-exclusion chromatography

Size-exclusion high-performance liquid chromatography (SEC-HPLC) was carried out in a 1200 Series HPLC system (Agilent Technologies, Santa Clara, CA, USA) and a Superdex® Peptide 10/300 ​GL column (30 ​× ​10 ​cm, 13 ​μm avg. size particle) (GE Healthcare, Uppsala, Sweden). Samples were eluted under an isocratic flow of 0.4 ​mL/min of 100% phosphate buffered saline (0.138 ​M NaCl, 0.0027 ​M KCl, pH 7.4) (Neogen® Corporation, MI, USA) at a pressure of 400 ​bar. The sample volume was 15 ​μL (1 ​mg protein/mL), and the analysis was performed at 220 ​nm. All samples were performed in triplicate.

### 2.7 Amino acid analysis

The amino acid analysis of the different samples was conducted in accordance with the Agilent method (Long, 2015). Briefly, 4 ​mg of protein of undigested OF, OPC and COPC, and 1.0 ​mL of digestates were hydrolyzed with 6 ​N HCl containing 0.1% phenol and Norvaline (as internal standard) for 24 ​h ​at 110 ​°C in glass tubes sealed under vacuum. The hydrolyzed samples were cooled to room temperature and solutions evaporated with nitrogen until dryness. Once dry, the amino acids were dissolved by the addition of 10 ​mM sodium borate buffer (pH 8.2, containing 0.1% (w/v) HCl) and then filtered with 0.22 ​μm PVDF filters (low protein binding) (Sigma-Aldrich, USA). Analysis was performed using an Agilent Poroshell HPH-C18 reversed-phase column monitored with Agilent 1200 series HPLC system (Agilent Technologies Canada Inc., Mississauga, ON, Canada), utilizing an automatic post-column OPA and 9-fluorenylmethoxycarbonyl group (FMOC) derivatization and detection using absorbance at 338 ​nm. The separation was performed at a flow rate of 1.5 ​mL/min, employing a mobile phase of A: 10 ​mM Na_2_HPO_4_, 10 ​mM Na_3_B_4_O_7_, 5 ​mM NaN_3_, adjusted to pH 8.2 with HCl and B: ACN:MeOH:water (45:45:10, v/v/v). The elution program was as follows: 0 ​min, 2% B, 1.0 ​min, 2% B, 20 ​min, 59% B, 25 ​min, 2% B.

The content of tryptophan in the samples (OF, OPC and COPC) was determined separately by alkali hydrolysis following the method of Yust et al. (2004), with slight modifications. Samples (15 ​mg of protein) were dissolved in 3 ​mL of 4 ​N NaOH, sealed in hydrolysis tubes and incubated in an oven at 110 ​°C for 24 ​h. Hydrolysates were cooled down, neutralized to pH 7 using 12 ​N HCl and diluted to 25 ​mL with 1 ​M sodium borate buffer (pH 9). Aliquots of these solutions were filtered through a 0.45 ​μm PVDF filters and then injected into a Nova-Pack C18 column (Waters, Mississauga, ON, Canada). An isocratic elution system consisting of 25 ​mM sodium acetate, 0.02% sodium azide (pH 9)/acetonitrile (91:9) delivered at 1 ​mL/min was used. Five standard mixture ampoules (containing 17 amino acids) at different concentrations (10 pM/μL to 1 ​nM/μL) were purchased from Agilent (Agilent Technologies Inc., Mississauga, ON, Canada) and used for the construction of the calibration curves. The elution times of each amino acid in analyzed samples were compared to those of the standard, and the amount of each amino acid was calculated in mg/g based on the peak area.

### 2.8 Determination of nutritional parameters

#### 2.8.1 *In vitro* protein digestibility-corrected amino acid score

An *in vitro* protein digestibility assay was also performed on each sample provided (Hsu et al., 1977; Tinus et al., 2012). Initially, the equivalent of 62.5 ​mg of protein of each test sample (OF, OPC and COPC) was added to 8 ​mL Milli-Q water and heated to 37 ​°C, and the pH of the solution was adjusted to 8.0. Simultaneously, a multi-enzyme cocktail including 3.1 ​mg/mL chymotrypsin (bovine pancreas ≥40 U/mg protein), 1.6 ​mg/mL trypsin (porcine pancreas 13,000–20,000 BAEE U/mg protein) and 1.3 ​mg/mL protease (*Streptomyces griseus* ≥15 U/mg solid) was prepared in 10 ​mL Milli-Q water, heated to 37 ​°C and adjusted to pH 8. A 1 ​mL portion of the enzyme cocktail was transferred to the sample solution, and the resultant pH drop was recorded every 30 ​s for 10 ​min. The *in vitro* protein digestibility (IVPD) was calculated as follows, where the ΔpH_10min_ is the change in pH in 10 ​min from the initial pH of about 8.0:

$$\text{IVDP\%}=65.66+18.10\times {\text{pH}}_{10\text{min}}$$

Meanwhile the *in vitro* protein-digestibility corrected amino acid score (PDCAAS) was calculated as a product of the amino acid score and IVPD% (Nosworthy et al., 2017).

#### 2.8.2 Determination of nutritional parameters

a) Amino acid score (AAS) was calculated using the FAO/WHO/UNU (FAO/WHO/UNU, 1985) reference pattern and using the following equation:

$$\text{AAS}=\frac{\text{mg ​of ​amino ​acids ​in ​}1\text{ ​g ​total ​protein ​}}{\text{mg ​of ​amino ​acids ​in ​requirement ​pattern}}\times 100$$

b) Essential amino acid index (EAAI) was calculated using the amino acid composition of a standard (whole egg protein) (Amza et al., 2013):

$$\text{EAAI}=\sqrt[{9}]{\frac{\left(\text{Lys}\times \text{Thr}\times \text{Val}\times \text{Met}\times \text{Ile}\times \text{Leu}\times \text{Phe}\times \text{His}\times \text{Trp}\right)\text{a}}{\left(\text{Lys}\times \text{Thr}\times \text{Val}\times \text{Met}\times \text{Ile}\times \text{Leu}\times \text{Phe}\times \text{His}\times \text{Trp}\right)\text{b}}}$$

where “a” represents the content of amino acids in test sample and “b” the content of the same amino acids in the standard (%).

c) Predicted biological value (BV) was calculated according to Amza et al. (2013) ​using the following equation:

BV=1.09(EAAI)−11.7

d) Protein efficiency ratio (PER) values were obtained from the amino acid composition of oat samples based on the following five equations (Amza et al., 2013):

PER ​1=−0.684+0.456(Leu)−0.047(Pro)

PER ​2=−0.468+0.454(Leu)−0.105(Tyr)

PER ​3=−1.816+0.435(Met)+0.780(Leu)+0.211(His)−0.944(Tyr)

PER ​4=0.08084(Thr+Val+Met+Ile+Leu+Phe+Lys)−0.1094

PER ​5=0.06320(Thr+Val+Met+Ile+Leu+Phe+Lys+His+Arg+Tyr)−0.1539

### 2.9 Antioxidant *in vitro* bioactivity

#### 2.9.1 Reducing power

Reducing power was determined according to Oyaizu (1988) ​with modifications. Samples (50 ​μL, 0.1 ​mg protein/mL) were added to 50 ​μL 0.2 ​M phosphate buffer (pH 6.6) and 50 ​μL 1% K_3_Fe(CN)_6_ and incubated with shaking at 50 ​°C for 20 ​min. Plates were incubated for another 10 ​min ​at 50 ​°C after addition of 50 ​μL 10% TCA and 10 ​μL 0.1% FeCl_3_, and absorbance was read at 700 ​nm using a microplate reader (BioTek Instruments, VT, USA). Results were expressed as absorbance at 700 ​nm.

#### 2.9.2 Trolox equivalent antioxidant capacity (TEAC) assay

The ABTS radical cation (ABTS^•+^) discoloration assay was performed according to Re et al. (1999) ​with some modifications. The ABTS^•+^ was produced by reaction of ABTS (7 ​mM in water) with potassium persulfate (2.45 ​mM final concentration) in the dark at room temperature for more than 12 ​h. Prior to the assay, the solution was diluted in EtOH and equilibrated at room temperature to give an absorbance of 0.70 ​± ​0.02 ​at 734 ​nm. A Trolox standard curve (0–700 ​μM of Trolox/mL) was prepared in EtOH. Samples (20 ​μL, 0.1 ​mg protein/mL) were incubated for 6 ​min with 220 ​μL of ABTS^•+^ radical cation solution before measuring absorbance at 734 ​nm. The TEAC was expressed as μM of Trolox equivalents/μg of protein.

#### 2.9.3 DPPH radical scavenging activity

Antioxidant capacity was also measured using the free DPPH radical method according to Liao et al. (2012) ​with modifications. Samples of 125 ​μL (0.1 ​mg protein/mL) were placed with 125 ​μL of 0.1 ​mM of DPPH solution (0.0394 ​μg/μL MeOH) per well, mixed for 30 ​min ​at room temperature and read at 517 ​nm. The DPPH scavenging activity was calculated into a Trolox curve (0–1000 ​μM of Trolox/mL), and results were expressed as μM of Trolox equivalents/μg of protein.

#### 2.9.4 Iron chelating activity

Fe^2+^ chelating activity was determined by measuring the formation of the Fe^2+^ ferrozine complex (Carter, 1971). Volumes of 25 ​μL of each sample (0.1 ​mg protein/mL) were mixed with 225 ​μL of buffer solution and 30 ​μL FeCl_2_ (0.01%, w/v). Ferrozine (12.5 ​μL, 40 ​mM) was added after incubation for 10 ​min ​at room temperature. Binding of Fe(II) ions to ferrozine generates a colored complex that was measured at 562 ​nm using a microplate reader (BioTek Instruments, VT, USA). Iron chelating activity was calculated as:

$$\%\;Chelating\;activity=\frac{ABS_{control}-ABS_{sample}}{ABS_{control}}\times 100$$

where ABS_control_ is the absorbance of the control and ABS_sample_ is the absorbance of the sample.

#### 2.9.5 Copper chelating activity

Cu^2+^ chelating activity was determined according to Saiga et al. (2003). Sodium acetate buffer, pH 6 (290 ​μL, 50 ​mM), 6 ​μL 4 ​mM pyrocatechol violet prepared in the same buffer, and CuSO_4_•_5_H_2_O (10 ​μg) were added to the hydrolysates (20.5 ​μL, 0.1 ​mg protein/mL). Absorbance at 632 ​nm was measured using a microplate reader (BioTek Instruments, VT, USA). Copper chelating activity was calculated as described above for iron.

#### 2.9.6 β-carotene bleaching method

Antioxidant activity was determined by measuring inhibition of β-carotene bleaching as described by Braca et al. (2001), with modifications. The reagent consisted of 1 ​mL β-carotene (2 ​mg/mL in chloroform), 20 ​mg of linoleic acid and 100 ​mg of Tween 20, that were vigorously mixed by vortexing and flushed with nitrogen gas in order to remove chloroform before addition of oxygen-sparged distilled water (100 ​mL). Samples (50 ​μL, 0.5 ​mg/mL protein) were added to 200 ​μL of the β-carotene reagent and incubated in the dark at 40 ​°C for 60 ​min before determination of absorbance at 450 ​nm. Antioxidant activity (AA) was calculated according to the following equation (Al-Saikhan et al., 1995):

$$DR=\frac{Ln\frac{ABS_{0min}}{ABS_{60min}}}{60}$$

where DR is the degradation rate among the absorbance at the beginning (ABS_0min_) and the end of the reaction (ABS_60min_) for blank, control and samples. Using the DR, the antioxidant activity (AOx) was calculated as % inhibition relative to the control as:

$$\%\;Antioxidant\;activity\left(AA\right)=\left(\frac{DR_{control}-DR_{sample}}{DR_{control}}\right)\text{x}100$$

where DR_control_ is the degradation rate of β-carotene in the absence of sample. Results were expressed as percentage (%) antioxidant activity.

#### 2.9.7 Scavenging activity by ORAC assay

The oxygen radical absorbance capacity (ORAC) assay was performed as previously described (Huang et al., 2010). The decreasing fluorescein (0.08 ​μM) absorbance by AAPH^•^ (150 ​mM) at 37 ​°C was recorded at 5-min intervals over 60 ​min using a BioTek™ fluorescent multi-mode microplate reader (Sinergy HTX, BioTek Instruments, VT, USA) with fluorescence filters (excitation 485 ​nm, emission 528 ​nm). Potassium phosphate buffer (pH 7.4, 75 ​mM) was used to dissolve samples (0.1–0.2 ​mg protein/mL) or Trolox standards (0–100 ​μM). Data analysis was done using Gen5™ software (Fisher Scientific, Nepean, ON, Canada). Results were expressed as μM Trolox equivalent/μg of protein.

### 2.10 Statistical analysis

Data were reported as mean ​± ​standard deviation of triplicate assays. Results were subjected to one-way ANOVA using Fisher's least significant difference (LSD) with a *p* ​< ​0.05 in SigmaPlot Version 13.0 software (Systat Software Inc., San Jose, CA, USA).

## 3 Results and discussion

### 3.1 Proximate composition

The proximate composition of OF, OPC and COPC is shown in Table 1. The protein content of the OF increased from 15.85 to 87.24 ​g/100 ​g dw for the OPC. Also, the cooking treatment did not affect the protein amount in the sample. The Navarro oat flour showed a higher protein content of 11–13 g/100 ​g dw compared to those reported for red oat (*A. byzantina*) and common oat (*A. sativa*) flours, the parental varieties of this hybrid genotype (Biel et al., 2009; Beloshapka et al., 2016; Youssef et al., 2016). The total CHO content in oat flour was 73.21%, but after A-APE (aqueous-alkaline protein extraction) followed by IEP (isoelectric precipitation) it decreased significantly (*p* ​< ​0.05) to 3.92% and remained constant after cooking (4.44%).

**Table 1:** Proximate composition of OF (oat flour), OPC (oat protein concentrate) and COPC (cooked oat protein concentrate).

**SAMPLE**	**PROTEIN (%)**	**FAT (%)**	**MOISTURE (%)**	**ASH (%)**	**CHO∗ (%)**
OF	15.83 ​± ​0.20^b^	7.92 ​± ​0.22^a^	0.93 ​± ​0.02^b^	2.11 ​± ​0.01^b^	73.21 ​± ​0.11^a^
OPC	87.24 ​± ​4.84^a^	4.44 ​± ​0.00^c^	1.00 ​± ​0.02^a^	3.40 ​± ​0.02^a^	3.92 ​± ​0.53^b^
COPC	86.59 ​± ​0.93^a^	4.54 ​± ​0.01^b^	0.97 ​± ​0.08^ab^	3.46 ​± ​0.08^a^	4.44 ​± ​0.43^b^

CHO∗, Carbohydrates calculated by difference. Results are reported in dry weight (dw) and presented as mean ​± ​S.D. (*n* ​= ​3). Different letters in same column indicate significant differences (*p*˂0.05).

### 3.2 SDS-PAGE of proteins

**Fig. 1:**
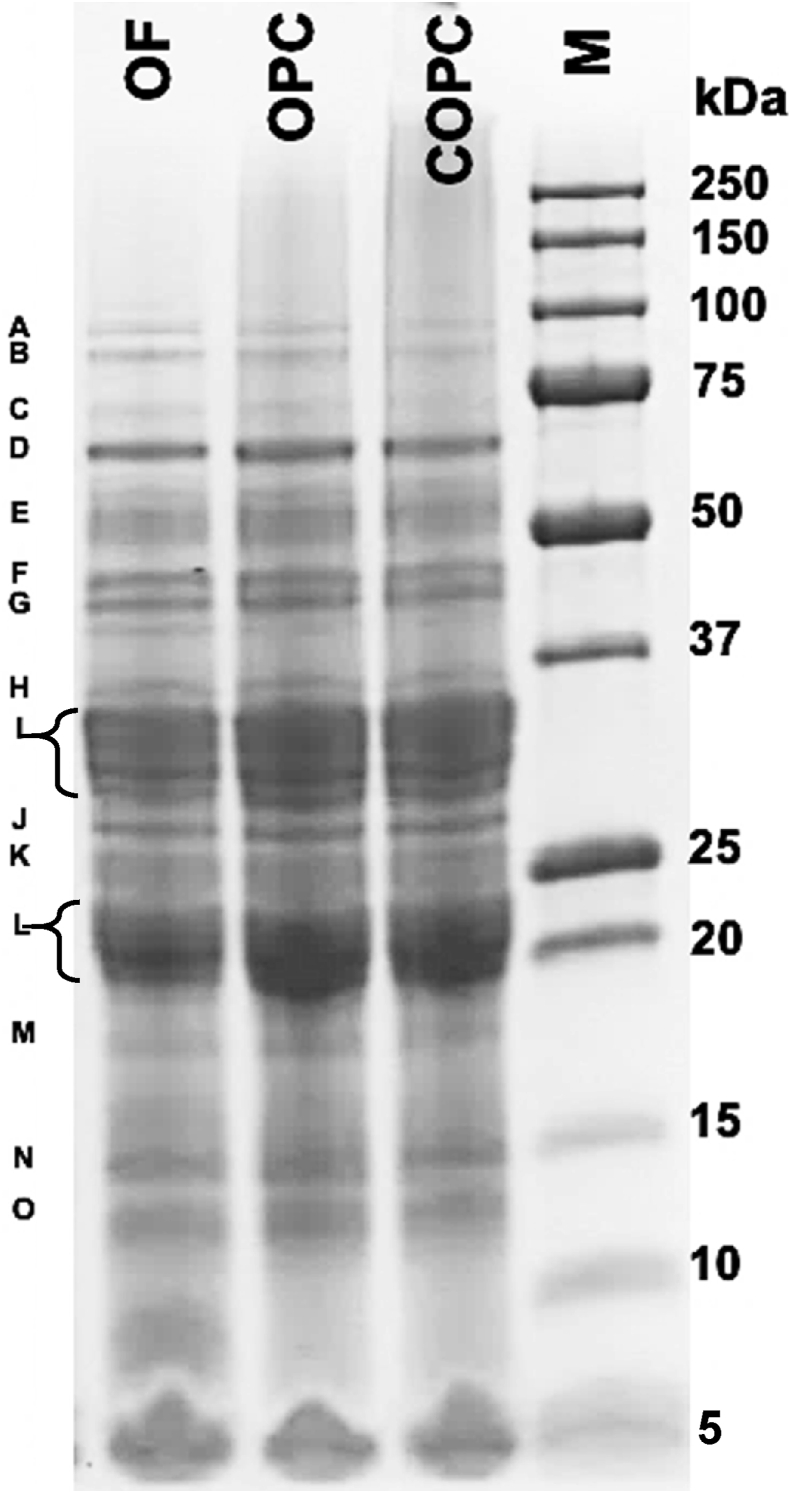
SDS-PAGE analysis of OF (oat flour), OPC (oat protein concentrate), COPC (cooked protein concentrate), under reducing conditions. Lanes M ​= ​MW marker (kDa). Protein bands: (A) 98 ​kDa: α-/β-albumin subunits; (B) 89 ​kDa: α-/β-albumin subunits; (C) 70 ​kDa: albumins; (D) 65 ​kDa: 7S globulins; (E) 52 ​kDa: 7S globulins; (F) 45 ​kDa: albumin subunits; (G) 40 ​kDa: albumin subunits; (H) 35 ​kDa: β-globulin subunits/avenins; (I) 30 ​kDa: β-globulin subunits/avenins; (J) 27 ​kDa: avenins; (K) 25 ​kDa: α-globulin subunits; (L) 22 ​kDa: α-globulin subunits; (M) 18 ​kDa: prolamin subunits; (N) 14 ​kDa: prolamin subunits; (O) 12 ​kDa: albumin subunits.

The electrophoresis of oat proteins under reducing conditions displayed a wide range profile of high-MW proteins between 12 and 98 ​kDa (Fig. 1). The protein profile of COPC showed no noticeable modifications after cooking conditions used in this study. A 70-kDa albumin band was detected with a visible high intensity, since this protein has been reported as one of the major protein groups, observed in several oat genotypes (Rossi and Luthe, 1983; Ercili-Cura et al., 2015; Sunilkumar et al., 2017). Oat globulins are bands above 50 ​kDa (Sunilkumar et al., 2017), two bands of 65 and 52 ​kDa were observed in the three oat samples, these are associated to 7S globulin fraction. Nieto-Nieto et al. (2014), reported that 7S globulins are polypeptides ranging around 55 ​kDa, and a small presence of a fraction with a MW of 65 ​kDa. Functional properties and physicochemical characteristics of oat proteins are strongly influenced by high-temperature processing (Mohamed et al., 2009). Furthermore, oat globulin has a thermal denaturation temperature of 110 ​°C, which is clearly higher than the temperature used in this study, thus not showing a greater impact on the major storage proteins (R Harwalkar and Ma, 1987). Compared to other proteins, globulins generally exhibit high denaturation temperatures, this has been attributed to strong hydrophobic interactions between subunits that strengthen at higher temperatures (Gorinstein et al., 1996). Zhao et al. (2004), studied the heat-induced aggregation of oat globulin, as a result of subjecting oat globulins upon heating at 100 ​°C, partial denaturation, dissociation into subunits and formation of soluble aggregates followed by formation of large insoluble aggregates may occur (60 ​min/110 ​°C), depending on the extent of thermal treatment (Zhao et al., 2004).

The presence of 89–98 ​kDa bands are related to α- and β-albumin subunits which are linked by disulfide bonds (under non-reducing conditions) (Ercili-Cura et al., 2015). A set of bands about 40–45 ​kDa could be identified as a group of albumin subunits (Ercili-Cura et al., 2015; Sunilkumar et al., 2017), which were detected in a similar intensity for the three oat protein samples, as well as β-globulin subunits (30–35 ​kDa). Avenins and other prolamins, which represent an average of 4%–15% of total proteins in oat cultivars, were found at 25–39 ​kDa (Sunilkumar et al., 2017). The α-subunits of globulins at 22–25 ​kDa were observed, as well as a group of prolamin subunits (14–18 ​kDa). Finally, at 12 ​kDa, another albumin subunit set was also detected, as reported by Ercili-Cura et al. (2015) ​and Sunilkumar et al. (2017).

The SDS-PAGE of oat samples revealed that the quality of oat proteins was not negatively affected by extraction and/or cooking processing. This rich diversity of proteins and protein subunits could suggest the potential health benefits of oat peptides on human health. Globulins, as the major proteins in oat, possess the highest number of bioactive peptides encoded within their parent sequences, such as antihypertensive and antidiabetic properties (Zheng et al., 2020), but also antioxidant properties (Ma et al., 2020), as well as avenins, being the only allergenic proteins present in oat (Vanvi and Tsopmo, 2016).

### 3.3 SEC-HPLC of OPC and COPC digestates

**Fig. 2:**
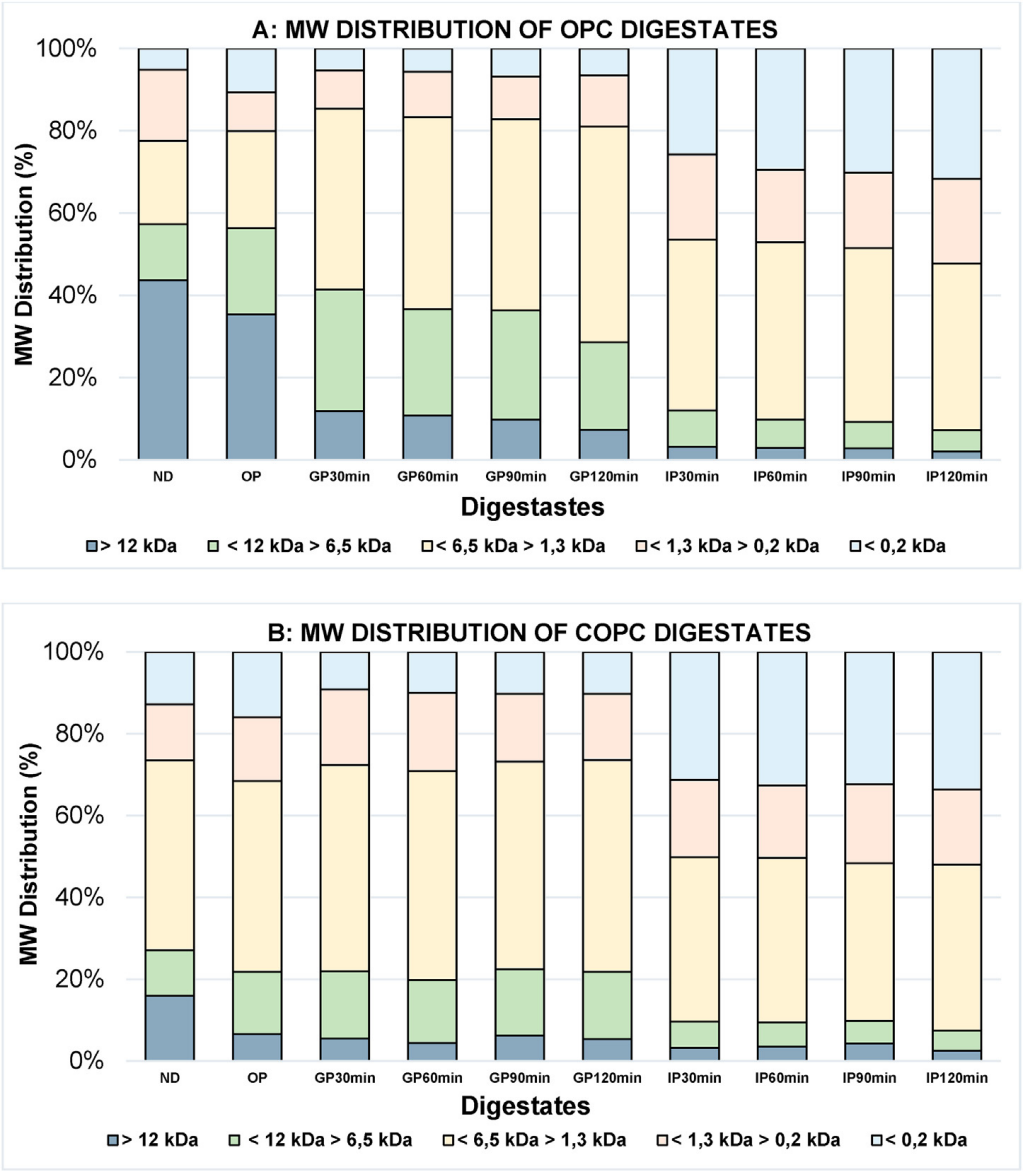
Molecular weight (MW) distribution of OPC (A) and COPC (B) digestates. ND: Non-digested sample; OP: Oral phase; GP: Gastric phase; IP: Intestine phase.

Fig. 2 shows the MW distribution of OPC (Fig. 2A) and COPC (Fig. 2B) through GID. In ND OPC, the molecules >12 ​kDa represented the highest proportion with 43.70%, while in COPC it was just 15.94%; meanwhile, in ND COPC, most of the proteins (46.33%) ranged between <6.5 and >1.3 ​kDa. Before digestion, it is clear that cooking had an effect on the MW distribution. Then during GP, OPC molecules of >12 ​kDa decreased to 35.33%, and increased up to 6.57% for COPC. Afterwards, free amino acids and/or dipeptides of <0.2 ​kDa represented >10% in both samples. During GP, the <6.5 to >1.3 ​kDa oligopeptides and peptides increased from 44.02% to 52.40% in OPC, but these were also >50% in COPC at this stage. At the IP, the >12 ​kDa represented <3.5% in both samples; the <6.5 to >1.3 ​kDa oligopeptides/peptides fractions were 40%–44% in OPC and 38%–41% in COPC. The <12.0 to >6.5 ​kDa fractions decreased to 5.18% and 4.93% in OPC and COPC, respectively. Meanwhile, the <1.3 to >0.2 ​kDa small peptides fractions ranged between 9.41% and 20.67% in OPC and 13.23% and 19.22% in COPC.

From the beginning to the end of GID, peptides of <6.5 ​kDa went from 42.72% to 92% of total peptide content, in which <1.4 ​kDa peptides are mostly digested peptides (∼50%). This evidenced the gradual hydrolysis of proteins by GID enzymes. However, some of these released peptides could remain intact by exo- and endoproteases present in the brush border membrane (Zhang et al., 2018). Small peptides of 4-16 amino acid units with molecular weight of 0.4–2 ​kDa have shown antioxidant properties (Görgüç et al., 2020). Several studies have found that short peptides are easily absorbed using Caco-2 ​cell models after GID and exert biological effects such as antioxidant and metal-chelating activities (Zhang et al., 2018).

### 3.4 Amino acid profile

The amino acid composition of OF, digested and non-digested OPC and COPC is summarized in Table 2. The relative amount of essential amino acids (EAA) in OF was 32.23%, but it statistically (*p* ​< ​0.05) increased to 36.40% after A-APE and to 62.88% after GID (OPC-IP_120min_). The EAA of ND COPC was 42.77% and did not show statistical differences (*p* ​< ​0.05) with COPC-IP_120min_ (47.19%). Moreover, the proportion of Lys (first limiting amino acid) is also influenced by A-APE, cooking and digestion, since Lys showed a proportion of 3.96% in OF, 7.29% in OPC-IP_120min_ and 6.39% in COPC-IP_120min_ (Supplementary Table 1). Meanwhile Thr, being the second limiting amino acid in oat, represented 3.85% in OF, 3.92% in OPC-IP_120min_ and 2.61% in COPC-IP_120min_. Thermal (87–110 ​°C) and enzymatic hydrolysis have a major impact on albumins (rich in limiting amino acids and EAA), but not on globulins (poor in EAA) (Klose, 2012; Mäkinen et al., 2017; Gorinstein et al., 1996). Outstanding enhancements in EAA of COPC-IP_120min_ were also observed for His, Met and Cys contents with 381%, 510% and 266%, respectively, in comparison with the EAA values from OF.

**Table 2:** Amino acids groups in OF (oat flour), OPC (oat protein concentrate) and COPC (cooked oat protein concentrate).

**SAMPLES**		**AMINO ACIDS (g/100 ​g protein)**				
		**EAA**	**PCAA**	**Aromatic**	**Hydrophobic**	**Sulphur**
Oat flour (OF)		32.23 ​± ​1.21^i^	11.53 ​± ​0.11^g^	8.72 ​± ​0.71^f^	26.17 ​± ​1.01^g^	2.34 ​± ​0.20^g^
**Oat Protein Concentrate (OPC)**	ND	36.40 ​± ​1.23^h^	17.03 ​± ​2.05^e^	10.46 ​± ​1.37^ef^	30.17 ​± ​2.10^f^	2.19 ​± ​0.23^g^
	OP	35.10 ​± ​2.14^h^	18.72 ​± ​0.97^e^	10.99 ​± ​1.41^ef^	29.27 ​± ​3.02^fg^	2.45 ​± ​0.54^g^
	GP_30min_	66.96 ​± ​3.55^ab^	26.76 ​± ​1.52^b^	18.06 ​± ​1.11^a^	50.52 ​± ​3.94^a^	4.86 ​± ​0.22^c^
	GP_60min_	70.74 ​± ​3.12^a^	28.83 ​± ​1.58^a^	18.72 ​± ​1.21^a^	53.34 ​± ​2.41^a^	5.31 ​± ​0.31^b^
	GP_90min_	67.59 ​± ​4.34^a^	27.36 ​± ​1.53^ab^	18.12 ​± ​0.52^a^	50.76 ​± ​2.62^a^	5.01 ​± ​0.30^bc^
	GP_120min_	68.28 ​± ​4.22^a^	27.36 ​± ​1.67^ab^	18.18 ​± ​0.98^a^	51.60 ​± ​2.09^a^	5.04 ​± ​0.28^bc^
	IP_30min_	61.44 ​± ​2.10^b^	24.87 ​± ​1.48^c^	16.65 ​± ​0.71^b^	46.41 ​± ​1.88^bc^	3.90 ​± ​0.17^e^
	IP_60min_	58.17 ​± ​3.19^b^	23.67 ​± ​1.44^cd^	15.54 ​± ​0.15^c^	44.04 ​± ​1.90^c^	3.87 ​± ​0.24^e^
	IP_90min_	59.10 ​± ​2.16^c^	23.73 ​± ​1.32^cd^	15.63 ​± ​1.01^bc^	44.76 ​± ​1.91^c^	3.99 ​± ​0.38^e^
	IP_120min_	62.88 ​± ​2.09^b^	24.69 ​± ​1.01^c^	17.25 ​± ​0.29^ab^	47.34 ​± ​1.90^b^	4.80 ​± ​0.31^c^
**Cooked Oat Protein Concentrate (COPC)**	ND	42.77 ​± ​1.32^fg^	14.22 ​± ​1.11^f^	10.10 ​± ​0.99^ef^	33.18 ​± ​2.43^e^	4.82 ​± ​0.44^c^
	OP	45.27 ​± ​2.17^f^	15.01 ​± ​1.51^f^	11.31 ​± ​0.89^e^	35.72 ​± ​2.31^de^	5.03 ​± ​0.54^bc^
	GP_30min_	34.47 ​± ​1.33^hi^	21.00 ​± ​2.06^d^	6.51 ​± ​0.51^g^	29.64 ​± ​2.01^f^	4.56 ​± ​0.31^cd^
	GP_60min_	39.48 ​± ​1.45^g^	17.91 ​± ​2.00^ef^	9.24 ​± ​0.86^f^	29.97 ​± ​2.00^f^	3.39 ​± ​0.30^f^
	GP_90min_	39.84 ​± ​1.64^g^	17.82 ​± ​1.01^ef^	9.72 ​± ​0.73^f^	29.55 ​± ​2.03^fg^	3.54 ​± ​0.31^f^
	GP_120min_	41.28 ​± ​1.39^g^	26.37 ​± ​1.99^b^	8.85 ​± ​0.97^f^	36.36 ​± ​2.31^e^	4.68 ​± ​0.21^cd^
	IP_30min_	59.91 ​± ​2.31^bc^	26.22 ​± ​1.01^b^	14.01 ​± ​1.02^d^	47.25 ​± ​2.55^b^	4.62 ​± ​0.22^d^
	IP_60min_	55.77 ​± ​2.06^d^	25.05 ​± ​1.13^bc^	14.61 ​± ​1.13^cd^	43.02 ​± ​3.01^c^	3.84 ​± ​0.27^e^
	IP_90min_	48.66 ​± ​1.64^e^	29.97 ​± ​1.52^a^	11.67 ​± ​1.08^e^	40.05 ​± ​3.04^d^	4.53 ​± ​0.33^cd^
	IP_120min_	47.19 ​± ​1.78^ef^	27.03 ​± ​1.00^a^	10.65 ​± ​1.00^ef^	40.78 ​± ​3.21^d^	7.80 ​± ​0.29^a^

ND: non-digested sample; OP: oral phase; GP: gastric phase; IP: intestinal phase. EAA (Essential AA): Phe ​+ ​His ​+ ​Ile ​+ ​Lys ​+ ​Thr ​+ ​Val ​+ ​Leu ​+ ​Trp ​+ ​Met ​+ ​Cys; PCAA (Positively charged AA): Arg ​+ ​Lys ​+ ​His; Aromatic: Phe ​+ ​Tyr ​+ ​Trp; Hydrophobic: Ala ​+ ​Ile ​+ ​Leu ​+ ​Val ​+ ​Gly ​+ ​Pro ​+ ​Phe; Sulphur (Sulphur-containing amino acids): Met ​+ ​Cys. Different letters in same column indicate significant differences (*p*˂0.05) under Fisher's test.

Positively-charged amino acids (PCAA) represented 13.41%, 13.67% and 14.22% in OF, OPC and COPC, respectively. However, after IP_120min_, OPC and COPC showed significant (*p* ​< ​0.05) increase of PCAA (19.53% in OPC and 14.22% in COPC). In this case, neither the protein extraction and/or cooking processes had an effect on the increase of PCAA in the protein concentrates. However, GID had a significant impact on PCAA due to proteolysis and thus an increased exposure of these amino acids (Udenigwe and Aluko, 2011). Structural modifications during enzymatic hydrolysis increased the accessibility to R-groups of PCAA, which have a negative influence in reducing antioxidant power and radical scavenging activity (Espinosa-Paez et al., 2017).

Aromatic amino acids did not show statistical (*p*>0.05) changes due to A-APE and cooking, as the values for OF, OPC and COPC represented about 8.72%, 10.46% and 10.10%, respectively. Even in COPC-IP_120min_, this value was 10.65%. Phe and Trp decreased with protein extraction and cooking, but Tyr contributed to keeping the aromatic amino acids proportion after GID. Aromatic amino acids are also non-polar and could play an important role in peptide polarity and bioactivity, particularly of oat glutelins (Ma et al., 2020).

Hydrophobic amino acids represented 26.17% in OF and 30.17% in OPC, while the hydrothermal process increased it statistically (*p* ​< ​0.05) to 33.18% in COPC. OPC-IP_120min_ and COPC-IP_120min_ showed values of 47.34% and 40.78%, respectively. It could be mainly due to the increment of Gly content (314%). The hydrophobic amino acids play a major role in antioxidant activity of DPPH and H_2_O_2_ radicals (Udenigwe and Aluko, 2011; Espinosa-Paez et al., 2017).

Sulphur-containing amino acids in OF, OPC and COPC represented 2.34%, 2.19% and 4.82%, respectively. The GID positively influenced the proportion of sulphur amino acids, since in OPC-IP_120min_ it was 3.80%, while in COPC-IP_120min_ it significantly (*p* ​< ​0.05) increased up to 7.72%. Thus, cooking followed by GID promoted an increase of Met and Cys. These amino acids have a central role in antioxidant activity (e.g. H_2_O_2_-scavenging activity and superoxide radical scavenging activity) (Udenigwe and Aluko, 2011).

The nutritional quality and bioactive properties of oat peptides depend on their amino acid profile (Mäkinen et al., 2017; Youssef et al., 2016). According to available data, Glu (25%) is the most abundant non-essential amino acids in oat food products, followed by Asp (>8%), and the EAA Leu (7%) (Mäkinen et al., 2017). This agrees with our results, but after GID, the highest values consist not only of these amino acids, but also of Arg, Phe and Lys with contents of about 6%–13% of total amino acids. These last EAAs were reported as limiting EAA in unprocessed flour from naked oat grains (Biel et al., 2009), but our results indicate that Lys is increased after protein extraction and GID. Oat, like other cereals, is considered as a poor source of Lys and other EAA (Mäkinen et al., 2017; Biel et al., 2009; Youssef et al., 2016), but we demonstrated that during GID its content is higher than other non-limiting amino acids (e.g., Asp, Ser or Tyr). However, more studies are required to understand the behavior of individual amino acids and peptides released during GID, before and after absorption. For example, there are concerns related to the reduced bioavailability of some amino acids, such as Lys, that may be chemically transformed during the processing of foods (FAO/WHO/UNU, 1985). During GID, the complexity of food matrix and the cleaving activity of proteolytic enzymes, i.e., pepsin (on aromatic or dicarboxylic L-amino acids at Phe and Leu residues only, and Ala-Gln bonds) and trypsin (on C-terminal Arg and Lys residues), are the main responsible of the half-life of intake biopeptides (which is relatively short), compromising their bioaccessibility, bioavailability and bioactivity at intestinal epithelium level (Yap and Gan, 2020; Sun et al., 2019).

### 3.5 Nutritional parameters

The nutritional parameters of OF, OPC and COPC are shown in Table 3. The *in vitro* protein digestibility (IVPD%) of OF was 85.13%, which was statistically (*p* ​< ​0.05) increased to 96.34% after protein extraction, but then decreased to 92.78% in COPC. Our results were higher than those for flours from Fabaceae genera seeds (80.71%–84.10%) (Pastor-Cavada et al., 2014). The highest PDCAAS obtained was 0.60 for OPC and showed no statistical difference (*p*>0.05) after cooking (0.57). The PDCAAS of OPC and COPC presented higher values than those reported for four oat cultivars (PDCAAS 0.41 to 0.52) (Pedó et al., 1999) and similar values to the recommended PDCAAS for children aged 6 months to 3 years (PDCAAS 0.58) (Abelilla et al., 2018). This increase might be due to protein extraction as through the concentration or isolation of the proteins. This will also impact the amino acid composition and digestibility of the protein and thus the protein quality.

**Table 3:** Nutritional parameters of OF (oat flour), OPC (oat protein concentrate) and COPC (cooked oat protein concentrate).

	**OF**	**OPC**	**COPC**
**IVPD%**	85.13 ​± ​0.82^c^	96.34 ​± ​0.30^a^	92.78 ​± ​0.43^b^
**PDCAAS**	0.51 ​± ​0.01^b^	0.60 ​± ​0.03^a^	0.57 ​± ​0.02^a^
**AAS%**	115.22 ​± ​3.24^a^	115.51 ​± ​4.96^a^	120.71 ​± ​4.96^a^
**EAAI%**	44.55 ​± ​4.09^b^	45.92 ​± ​4.10^b^	88.14 ​± ​13.13^a^
**BV%**	36.86 ​± ​0.23^c^	38.35 ​± ​1.91^b^	84.37 ​± ​6.74^a^
**PER 1**	2.37 ​± ​0.01^b^	2.89 ​± ​0.21^a^	2.70 ​± ​0.13^a^
**PER 2**	2.25 ​± ​0.02^b^	2.74 ​± ​0.11^a^	2.58 ​± ​0.21^a^
**PER 3**	0.97 ​± ​0.02^b^	1.72 ​± ​0.26^a^	1.91 ​± ​0.22^a^
**PER 4**	2.09 ​± ​0.03^b^	2.31 ​± ​0.30^a^	2.42 ​± ​0.28^a^
**PER 5**	2.31 ​± ​0.02^b^	2.57 ​± ​0.27^a^	2.66 ​± ​0.31^a^

OF: oat flour; OPC: oat protein concentrate; COPC: cooked oat protein concentrate; IVPD: *in vitro* protein-digestibility; PDCAAS: protein digestibility corrected amino acid score; AAS: amino acid score; EAAI: essential amino acid index; BV: biological value; PER: protein efficiency ratio. Mean ​± ​S.D. same letters in a line indicate statistical difference (*p* ​< ​0.05) under Fisher's test.

The amino acid scores (AAS%) for OF, OPC, and COPC were 115.22%, 115.51% and 120.71%, respectively, with non-statistical differences between them (*p* ​< ​0.05). Our AAS% values were, at least two times higher than those reported for other oat cultivars (50.0–60.2%) (Abelilla et al., 2018) but ranged between those reported for rich protein-rich *Fabeae* seeds (100.15%–123.03%) (Pastor-Cavada et al., 2014; Parya Samaei et al., 2020). The essential amino acid index (EAAI%) for OF was 44.55% and 45.92% for OPC, showing no statistical differences (*p* ​< ​0.05). However, for COPC it was statistically higher (*p* ​< ​0.05) with 88.14%, and thus the heating step dramatically increased (>95%) the EAAI% of OPC. As mentioned previously, hydrothermal and enzymatic digestion have a drastic effect on albumins rich in EAA, but a poor effect on globulins with low contents of these amino acids (Klose, 2012; Mäkinen et al., 2017; Gorinstein et al., 1996).

Our results were higher than the values reported for several *Fabeae* beans (39.90%–41.75%) (Pastor-Cavada et al., 2014), but only COPC was higher than alcalase-trypsin hydrolyzed *Vicia faba* protein hydrolysates (69.95%–87.11%) and lower than individual Vicia faba protein hydrolysates produced with trypsin, pepsin and alcalase (88.34%–94.92%) (Parya Samaei et al., 2020). According to Amza et al. (2013), EAAI above 90% are assumed to be of good nutritional quality. However, COPC is close to this value, but it is necessary to evaluate this parameter on digested COPC. The biological values (BV%) of OF were 36.86%, 38.35% for OPC and 84.37% for COPC, being statistically different (*p* ​< ​0.01) between them. The values of OF and OPC fall into legume *Fabeae* values (26.40%–38.41%) (Pastor-Cavada et al., 2014), while COPC results are comparable to some faba bean protein hydrolysates (64.54%–91.76%) (Parya Samaei et al., 2020).

The theoretical protein efficiency ratio (PER), calculated using five equations, considers different amino acid contents (Amza et al., 2013). The PER results of the five equations showed that OF is statistically lower (*p* ​< ​0.05) than OPC and COPC. The PER values in OF ranged between 0.97 and 2.37, while OPC showed values between 1.72 and 2.89, and COPC showed 1.91–2.70. The PER is a quality index with values ranging from 0 (low protein quality) up to 2 and above (high protein quality) (Amza et al., 2013). The highest PER values found in OPC and COPC indicate that protein quality is enhanced after A-APE. Pastor-Cavada et al. (2014) ​obtained PER 1-PER 3 values of 2.44–3.07 for *Fabeae* genera seeds, while Parya Samaei et al. (Parya Samaei et al., 2020) reported PER 1-PER 5 values of 0.87–5.19 in faba bean enzyme hydrolysates. Thus, oat protein-enriched concentrates could be considered as a good-quality food protein source, rich in essential amino acids that could be used as an ingredient for the development of functional foods.

### 3.6 Bioactivity of OPC and COPC digestates

**Fig. 3:**
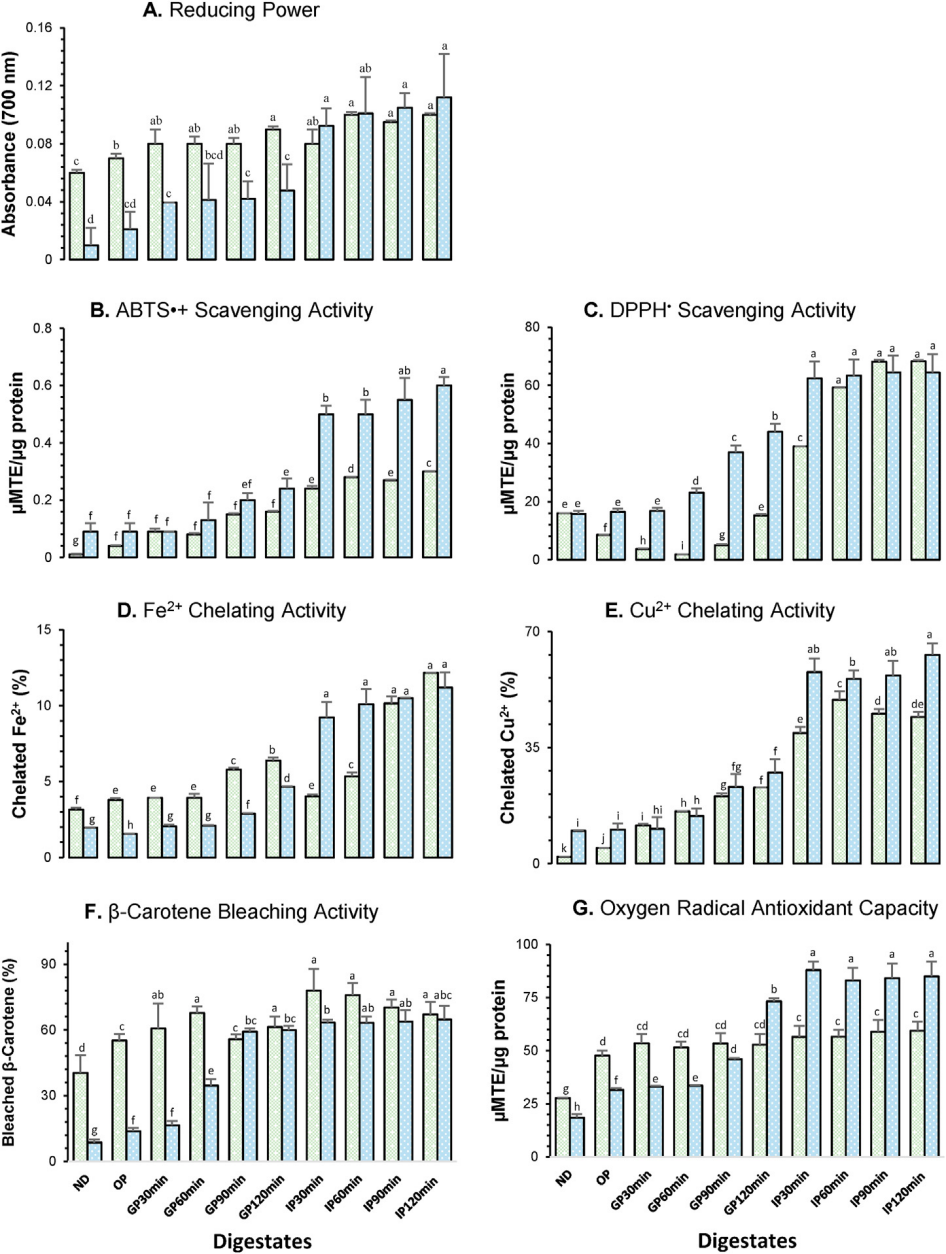
*In vitro* AOx bioactivity of OPC and COPC digestates. Light-green bars: oat protein concentrate; light-blue bars: cooked oat protein concentrate. ND: Non-digested sample; OP: Oral phase; GP: Gastric phase; IP: Intestinal phase. For Scavenging ABTS radical, Scavenging DPPH radical and ORAC assays: μMTE/μg protein, μM of Trolox equivalents/μg of protein, respectively. Same letters in the graphic indicate no statistical differences (*p* ​< ​0.05). (For interpretation of the references to color in this figure legend, the reader is referred to the Web version of this article.)

In this study, we also evaluated the impact on bioactivity of released OPC and COPC peptides during GID. Functional properties of oat peptides have a strong dependence on protein solubility and digestibility (Esfandi et al., 2019). The radical scavenging activity of plant food sources has been receiving more and more attention from researchers in recent years (Zhong et al., 2019; Ma et al., 2020; Zhang et al., 2018; Carrasco-Castilla et al., 2012a). The antioxidant effect of peptides is based on free-radical or reactive species scavenging activity on hydrogen atom transfer (HAT) and single-electron transfer (SET) (Udenigwe and Aluko, 2011) and generally it is attributed to 4-16 amino acid units with molecular weights between 0.4 and 2 ​kDa (Görgüç et al., 2020). The *in vitro* antioxidant activity of digested samples is presented in Fig. 3.

#### 3.6.1 Reducing power

Reducing power assay (Fig. 3A) was used to measure the reduction of the Fe^3+^-ferricyanide complex to the ferrous form by hydrolyzed peptides, related with formation of Perl's Prussian blue. The reducing power of OPC is significantly enhanced with digestion: after oral phase, the reducing power increases from 0.06 to 0.07 (an increase in the absorbance indicates an increase in the antioxidant activity). After that, it was increased in GP and not modified until IP_30min_. Finally, after IP_60min_, the reducing power was significantly higher than previous stages, and constant up to GID end.

On the other hand, the reducing power of ND COPC was 0.01, which was statistically increased (*p* ​< ​0.05) after GP_90min_ to 0.05. During IP, it was statistically stable (*p* ​< ​0.05) between 0.09 and 0.11. According to previous reports, in this assay the SET mechanism of antioxidant peptides is attributed to Tyr, Met, Cys, His and Lys peptide residues (Carrasco-Castilla et al., 2012b; Zhong and Shahidi, 2015; Wang et al., 2016) and these particular amino acids increased with protein extraction and GID (Table 1). Enzymatic hydrolysis of native plant proteins favored the Fe^3+^-ferricyanide complex synthesis (Parya Samaei et al., 2020). However, more studies on the peptide profile of oat digestates are required to better understand the possible interactions and/or mechanisms of oat peptides with ferrous compounds.

#### 3.6.2 TEAC assay

The ABTS decolorization assay is a suitable method to determine the antioxidant capacity of substrates that have the capacity to scavenge the ABTS^•+^ radical by their HAT ability, but also SET ability can be measured (Zhong and Shahidi, 2015). The balance of these two mechanisms is determined by the chemical structure of antioxidants and pH of the matrix (Zhong and Shahidi, 2015). The ND OPC sample showed a scavenging activity of 0.01 μMTE/μg of protein. This was significantly (*p* < 0.05) increased to 0.04 μMTE/μg of protein after oral phase. After 60 ​min of gastric digestion, the scavenging activity was duplicated and significantly (*p*<0.05) enhanced again at 90 ​min and 120 ​min, respectively (Fig. 3B). The AOx was further increased from 0.24 to 0.30 μMTE/μg, at 30–120 ​min ​at IP, showing no statistical differences (*p* < 0.05) between 60 and 90 ​min. The *in vitro* GID of OPC improves the scavenging activity on ABTS^•+^.

Moreover, the scavenged ABTS^•+^ in ND COPC showed values of 0.09 μMTE/μg protein, being statistically higher (*p* ​< ​0.05) than ND OPC. At GP_120min_ the scavenged ABTS^•+^ of gastro-digested samples was higher, with 0.24 μMTE/μg protein. However, during IP, the AOx of COPC showed exceptional values of 0.50 μMTE/μg protein at IP_30min_ to 0.60 μMTE/μg protein at IP_120min_. These data are close to other protein hydrolysates from vegetal and animal sources (Tagliazucchi et al., 2016; Carrasco-Castilla et al., 2012a; Wang et al., 2016), leading to conclusion that AOx measured by Trolox equivalent antioxidant capacity assay is enhanced throughout GID. Aromatic amino acids (Tyr, Phe) have the ability to donate protons easily to electron deficient radicals (Görgüç et al., 2020) whose contents were increased particularly in digested COPC. Ethanolic extracts of defatted oat flour from nine oat genotypes showed ABTS^•+^ values ranging from 0.825 to 3.528 ​mg of Trolox equivalents/mg (Brindzová et al., 2008), however, phenolic compounds, avenanthramides and β-glucan present in this extract may influence their AOx final values. Enzymatic hydrolysis of faba bean proteins showed an increase >1.5 fold of ABTS^•+^ scavenging activity in trypsin-pepsin hydrolysates, compared to individual hydrolysates of trypsin, pepsin or alcalase (Parya Samaei et al., 2020). This means that the combination of specific enzymes could improve AOx of peptides in protein-rich plant sources.

#### 3.6.3 DPPH scavenging activity

The DPPH^•^ scavenging activity of ND OPC was 15.98 μMTE/μg protein. This was statistically (*p*<0.05) higher than in the oral and GP stages, except at GP_120min_. After that, the scavenging activity of digestates continues increasing to 68.26 μMTE/μg protein at the end of GID (Fig. 3C). However, although the ND COPC did not show a statistical difference (*p* ​< ​0.05) from ND OPC, it remained steady until GP_30min_. At GP_60min_ the AOx of COPC was 23.11 μMTE/μg, which statistically increased (*p* ​< ​0.05) until IP_30min_ to 62.42 μMTE/μg, with no modifications through the whole GID. Peptides of both samples (OPC and COPC) enhanced their DPPH^•^ scavenging activity about 400% after GID.

According to Ma et al. (2020), alcalase hydrolyzed peptides of >10 ​kDa from oat globulins exhibited an increase of 24.5% in DPPH scavenging activity. In alcalase and trypsin hydrolysates from oat flour, Tsopmo et al. (2010) ​found an increase of between 25% and 35% in DPPH AOx, but they did not report statistical differences after 60 ​min of enzymatic digestion. Espinosa-Páez et al. (Espinosa-Paez et al., 2017) also observed an increase in DPPH^•^ scavenging ability in oat bio-processed samples after fungus fermentation followed by GID (INFOGEST method). However, it was enhanced by only 225%. In both cases, the enzymatic digestion increased the AOx, but this suggests that protein fractionation/purification steps could affect positively the DPPH^•^ scavenging activity of oat peptides. The DPPH scavenging activity could be associated with the increase of hydrophobic or aromatic amino acids (Udenigwe and Aluko, 2011).

#### 3.6.4 Iron chelating activity

The chelation of ferrous iron form (Fe^2+^) is an important antioxidant process, as Fe^2+^ can facilitate the production of reactive oxygen species (ROS) within human systems. Thus, the synthesis of ROS and pro-oxidant states into the human body can be prevented (Walters et al., 2018). The iron-chelating activities of OPC and COPC samples were determined by measuring the formation of the Fe^2+^-ferrozine complex (Fig. 3D). Oat protein content showed a Fe^2+^ of 3.16%, while it increased significantly (*p* < 0.05) to 3.80% during OP. After that, the Fe^2+^ chelating activity was constant at 3.94% up to GP_60min_. A 6.40% increase was observed at the end of GP. At IP_30min_, the chelation was significantly decreased to 4.03% and then increased to 5.35% after another 30 ​min of intestinal digestion. At the end of GID, an increasement 22.17% of Fe^2+^ chelating activity was observed. The iron-chelating activity of COPC was statistically inferior (*p* ​< ​0.05) in all cases before IP_30min_, with values between 1.97% and 4.65%. However, during IP these values were similar (*p* ​< ​0.05) between 9.24% and 11.22%, and at IP_120min_ the chelated Fe^2+^ was the same as that observed in OPC.

Histidine-containing peptides are associated with the HAT and metal ion-chelating ability of the imidazole group (Görgüç et al., 2020; Walters et al., 2018). Its content was increased in digested COPC, which could influence the chelating properties of digestates. The iron-chelating activity of oat peptides may be modified depending on the nature of proteins, digestion conditions and extraction procedure (Esfandi et al., 2019). For example, Baakdah & Tsopmo (Baakdah and Tsopmo, 2016) reported a Fe^2+^ chelating activity of 39.7% in oat bran protein hydrolysates using Protamex. Meanwhile, Esfandi et al. (2019) ​reported Fe^2+^ chelating activity of 38%–58% when oat bran proteins were extracted with a cellulase-protease sequential system and increased to between 40% and 79% when viscozyme-protease was used. These enzymes (carbohydrases) are not specific for proteins/peptides, but they could break hydrogen links that may release peptides from complex peptide structures (Esfandi et al., 2019).

#### 3.6.5 Copper chelating activity

Moreover, Cu^2+^, like Fe^2+^, is also a free-radical generator in biological systems promoting oxidative damage at different levels, capable of inducing the oxidation of lipids, proteins and DNA (Carrasco-Castilla et al., 2012a). The Cu^2+^ chelation ability increased constantly from 2.11% to 49.43% at IP_60min_ (Fig. 3E). Afterwards, it decreased significantly (*p*<0.05) to 45.26% to be constant until the end of GID. Before IP_90min_, digestates showed statistical differences (*p*<0.05) among them. Similar Cu^2+^ chelating behavior was observed for COPC. In this case, the chelation capacity was 9.90% in ND COPC and statistically constant until GP_60min_. At GP_120min_, the chelated Cu^2+^ was 27.50%, without statistical difference (*p*>0.05) with OPC at this stage. Afterwards, during the IP, Cu^2+^ chelation was estimated at between 57.8% and 63% without statistical changes (*p*>0.05) from IP_30min_ to IP_120min_. At the intestinal phase, the Cu^2+^ chelated is 29.9% higher in cooked samples in comparison with the OPC. This essential trace element is vital as enzyme cofactor and it is capable of binding to His, Cys and Met to allow the absorption and transport of amino acids in the human body (Antolovich et al., 2002). Increases in His, Cys and Met contents in our experiments could have directly affected the Cu^2+^ chelating activity. However, specific analysis on this topic is required. More studies of the chelating activity of copper and other transition metals on the impact of oat peptides produced during GID are necessary to better understand this important bioactivity (Carrasco-Castilla et al., 2012a; Baakdah and Tsopmo, 2016).

#### 3.6.6 β-carotene bleaching activity

The β-carotene antioxidant activity is a rapid colorimetric assay close to biological systems, able to lead the following antioxidant mechanisms: protecting oxidable linolenic acid, neutralizing oxidation products and protecting β-carotene from oxidable interactions with ROS (Gaetke and Chow, 2003). These multiple pathways of action can possibly exert antioxidant values over 100%, but it is challenging to attribute the AOx to one group of compounds or a single antioxidant mechanism (Carrasco-Castilla et al., 2012a). In this way, the β-carotene-bleaching activity of ND OPC was 40.39%, which is statistically lower (*p* ​< ​0.05) than oral phase, with 55.25% and GP with a maximum value of 67.75% (Fig. 3F). During IP, no statistical differences (*p*>0.05) were found with GP digestates. In contrast, the ND COPC showed %AA values of 8.67%, lower (*p* ​< ​0.05) than its counterpart. Then AOx increased at the oral phase to 13.86% and remained constant until GP_30min_. Afterwards, it improved to 59.23% up to the end of GP with no modifications (*p* ​< ​0.05) until IP_120min_.

#### 3.6.7 ORAC quenching activity

The ORAC assay evaluates the ability of a substance to scavenge the peroxyl radical (ROO•) (Huang et al., 2010). This radical is common in biological systems and is produced mainly by lipid oxidation. The ORAC, as well as β-carotene bleaching activity, is a relevant assay to simulate oxidant states under physiological conditions (Zhong and Shahidi, 2015). In this experiment, we observed that ROO• neutralizing activity increased with the protein digestion process, since in ND OPC, the ORAC value was 27.70 μMTE/μg protein, and it was significantly (*p*<0.05) increased at oral phase to 47.71 μMTE/μg protein, was enhanced again to 53.47 μMTE/μg protein at GP_30min_. Following GP_30min_, the ORAC value remained statistically constant (*p*<0.05) until the end of the stage (Fig. 3G). Finally, the highest ORAC highest value was at IP_120min_ with 59.41 μMTE/μg protein.

In COPC, the ND sample showed an ORAC value of 18.49 μMTE/μg protein, being 35% lower than for the ND OPC. However, after oral phase, the ORAC results indicated a statistical increase from 31.6 to 73.24 μMTE/μg protein at GP_120min_, which was higher than the maximum value observed in OPC. Digestive conditions, like pH changes, redox potential variability, or isoelectric point of fluids, could have been affected by AOx of peptides from OPC samples, but previous treatments (as cooking) may also influence the resulting bioactivity of peptides (Ashaolu, 2020). Moreover, during the IP, ORAC values reached 83.09–88.02 μMTE/μg protein in COPC without statistical changes (*p*>0.05) between them. The results obtained showed OPC AOx was enhanced more than two times at the end of GID.

The ROO• seems to be favored by the increase of Cys, Phe, Leu and Ile amino acid residues, as well as hydrophobic amino acids (Udenigwe and Aluko, 2011), whose content was increased with A-APE and GID (Table 1). An enriched content of HAT amino acids, such as His and Tyr, also contributes to the enhancement of AOx values by ORAC assay conditions (Ashaolu, 2020). Wattanasiritham et al. (2016) ​also reported improved ORAC values in trypsin-hydrolyzed rice bran. However, Vanvi & Tsompo (Vanvi and Tsopmo, 2016) found that, for oat pepsin hydrolysates, ORAC quenching ability was not related to the sample protein content, but rather corresponded to the oat proteins and peptides chemical structure and amino acid conformation. These last two studies and our results suggest that the ROO• quenching activity improved with enzymatic hydrolysis, meaning that ORAC values are enhanced with the release of small peptides, but contrary to these results, Tsopmo et al. (2010) ​reported that peptides about 2 ​kDa showed lower AOx in comparison to the whole hydrolysates.

Peptide digestates/hydrolysates are a complex mixture of large, medium and small peptide fractions that could provide valuable information on possible antioxidant mechanisms, depending on the methods assessed. Nevertheless, the free-radical scavenger action of bioactive peptides might be influenced by the ionization potential of the reactive functional group and the pH of the matrix (Ashaolu, 2020). It is important to mention that some phenolic compounds (e.g. ferulic and *p-*coumaric acid) could be still complexed to proteins and carbohydrates after alkaline extraction, therefore, during OP and IP, α-amylase may release a small fraction of bound phenolics and chelating free amino acids that might contribute to the observed AOx in samples (Multari et al., 2018). For all mentioned above, it is necessary to reach a consensus on the different *in vitro* antioxidant methods used to explain accurately the AOx mechanisms of bioactive peptides, before guaranteeing their effectiveness on human health.

## 4 Conclusions

To the best of our knowledge, the present work is the first report on the impact of cooking on oat protein nutritional parameters and antioxidant *in vitro* bioactivity using an *in vitro* simulated gastrointestinal digestion protocol (INFOGEST). The protein-extraction procedure and the cooking method showed no negative effects on protein composition and enhanced its nutritional quality (IVPD, PDCAAS, AAS, EAAI, BV and PERs), suggesting that these are high-protein-quality plant-food sources. Furthermore, A-APE followed by IEP allowed the elimination of potential interfering compounds and isolating/enriching protein in order to assess mainly the influence of bioactive peptides rather than other compounds present in the raw flour, as it is such a complex matrix. The GID of OPC and COPC progressively modified the peptide profile, increasing the <0.2 ​kDa of amino acids/dipeptides especially at the last stage of the intestinal phase, thus producing peptides with low MW and improved bioactivity. The amino acid profile showed an increase in essential, hydrophobic, positively-charged and aromatic amino acids, which directly influenced the antioxidant properties of the peptides produced during digestion. Throughout GID, depending on the gut enzymes specificity, a diversity of smaller peptides and free amino acids is generated, and thus changes in the length, concentration and composition of free amino acids and/or small–medium sized peptides affected the antioxidant activity measured. The peptides produced may even have a synergistic activity, helping somewhat to explain the results obtained in this study. Further purification studies should be carried out to identify the sequence of the peptides present at the intestinal stage, which are mainly responsible for the high bioactivities found. Additionally, the relationship between the structure and function of the peptides to elucidate the specific antioxidant mechanisms exerted should be taken into consideration. The results from this work could be useful for future research to develop food ingredients with tailor-made functionalities based on oat peptides with specific potential health promoting benefits.

## Compliance with ethical standards

The authors declare that they have no known competing financial interests or personal relationships that could have appeared to influence the work reported in this paper.

## Formatting of funding sources

Oscar Abel Sánchez-Velázquez's scholarship was provided by the Consejo Nacional de Ciencia y Tecnología (CONACYT) through doctoral scholarship 504305. This project was supported by A-base funding of AAFC (Project # J-00138).

## CRediT authorship contribution statement

**Oscar Abel Sánchez-Velázquez:** Conceptualization, Methodology, Investigation, Data curation, Writing - original draft. **Edith Oliva Cuevas-Rodríguez:** Supervision, Writing - review & editing. **Martin Mondor:** Validation, Writing - review & editing. **Sabine Ribéreau:** Methodology, Investigation. **Yves Arcand:** Validation, Resources, Data curation. **Alan Mackie:** Validation, Writing - review & editing. **Alan Javier Hernández-Álvarez:** Conceptualization, Supervision, Writing - review & editing.

## Declaration of competing interest

The authors declare that they have no known competing financial interests or personal relationships that could have appeared to influence the work reported in this paper.
